# Supplementary material for: Image restoration of degraded time-lapse microscopy data mediated by near-infrared imaging
Source: Nat Methods. 2024 Jan 4;21(2):311–21. doi: 10.1038/s41592-023-02127-z (PMC10864180; doi:10.1038/s41592-023-02127-z)
Supplement: Supplementary file 1 — Supplementary Notes 1–5, Supplementary Tables 1–3, Supplementary Figs. 1–11, Supplementary Videos 1–3 captions and supplementary references. [file 41592_2023_2127_MOESM1_ESM.pdf]

nature methods

Article https://doi.org/10.1038/s41592-023-02127-z

Image restoration of degraded time-lapse microscopy data mediated by near-infrared imaging

In the format provided by the authors and unedited

Supplementary Information to

Image restoration of degraded time-lapse microscopy data mediated by near-infrared imaging.

Nicola Gritti* [1,2], Rory M. Power* [1,3], Alyssa Graves [1], Jan Huisken [1,4,5,6]**

[1] Morgridge Institute for Research, 330 N Orchard St, Madison, WI 53715 USA
[2] European Molecular Biology Laboratory Barcelona, Carrer del Dr. Aiguader 88, 08003 Barcelona, Spain
[3] European Molecular Biology Laboratory Heidelberg, Meyerhofstrasse 1, 69117 Heidelberg, Germany
[4] Department of Integrative Biology, University of Wisconsin Madison, 250 N Mills St., Madison, WI 53706 USA
[5] Department of Biology and Psychology, Georg-August-University Göttingen, Friedrich-Hund-Platz 1, 37077 Göttingen, Germany
[6] Cluster of Excellence "Multiscale Bioimaging: from Molecular Machines to Networks of Excitable Cells" (MBExC), University of Göttingen, Germany

* These authors contributed equally.
** Corresponding author: jan.huisken@uni-goettingen.de

Supplementary Note 1:

Chromatic performance and calibration of the IR-mSPIM.

The IR-mSPIM corrects axial chromatic aberrations (i.e. a wavelength-dependent working distance) by motorizing the detection objective (optimized for multiphoton imaging in the NIR) and immersion chamber. Air objectives launch the light sheets through chamber windows, and so the light sheet position and, hence, the imaged section remains static when refocusing in this manner. The correct refocus position was determined by imaging fluorescent bead phantoms dispersed in agarose. A mixture of beads allowed excitation at all available laser wavelengths (405, 488, 561, 640, 685, 785, 808 nm). The phantom comprised TetraSpeck (excitable at 405, 488, 561, and 640 nm) and Degradex PLGA NIR (excitable at 640, 685, 785 and 808 nm) fluorescent beads. The correction thus determined corresponds to the pass-band of each emission filter rather than the laser line used for excitation. As such, the correction is valid for other fluorophores (e.g. GFP, AlexaFluor dyes) when using the same emission filters. The optimum working distance was determined from the maximum variance achieved for a defocus series for each laser/emission filter combination. The cross-excitability of the two fluorescent bead species used provided the basis for aligning the far-red/NIR lasers to the visible lasers (which are inherently co-aligned out of the single-mode fiber): The far-red/NIR lasers (685, 785, and 808 nm) are aligned by visualizing the same subset of beads as when using the 640 nm line, using the same emission filter (845/55 bp) in each case, hence decoupling the laser alignment from the chromatic aberration of the objective lens. Despite the refocus step, some axial misalignment of the individual colors is still possible

and may vary depending on the depth in tissue or the depth inside the agarose bead column for which the refocus calibration is made.

Moreover, the refocusing does not correct for lateral chromatic aberrations. Since the IR^2 training is reliant on the 1:1 correspondence between different color channels, residual misalignments must be corrected for. Any residual axial/lateral chromatic aberrations are accounted for via a registration step (see "Deep Learning" in Methods). As a demonstration, a two-color fluorescent bead sample is shown in Supplementary Figure 1. As shown in A–C, there is a noticeable misalignment between the two color channels. The misalignment is smaller in *z,* highlighting that the refocus scheme broadly corrects for axial color. Following registration, the beads are well aligned (D–E).

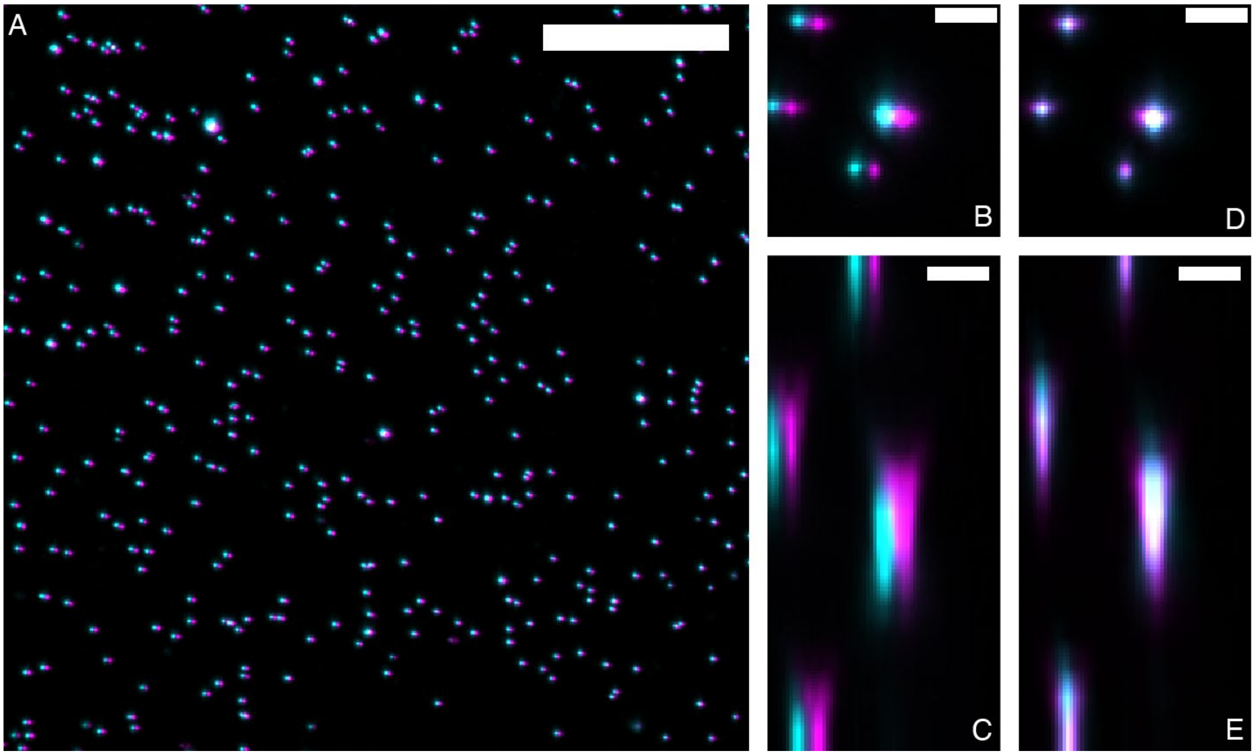

Supplementary Figure 1: Multi-color patch registration. A maximum intensity projection from a stack of fluorescent beads: cyan: 488 nm excitation, 525/50 nm emission (band center/full-width at half maximum bandwidth), magenta: 640 nm excitation, 697/60 nm emission. A: The center 200 µm × 200 µm of the full imaging field of view (scale bar = 50 µm). B–E one patch extracted from the full volume: 18.7 µm × 18.7 µm × 39.7 µm shown as maximum intensity projections over the excluded axis (scale bars: 5 µm). B/D *xy*-views C/E *xz*-views. B/C pre registration. D/E post registration.

The fluorescent beads used are sub-diffraction-sized, providing a route to explore the resolving performance of the IR-SPIM via the PSF. The scaling of lateral spatial resolution with wavelength would suggest that the performance is highly dependent on the specific emission band of the fluorophore and, hence, that the IR dyes would provide about half the resolving capability of GFP. However, SPIM often employs undersampling in the

imaging path (with respect to NA), to allow for a larger field of view while maintaining the light collection and axial resolution benefits of high NA. The data presented are captured at ca. 11.1× and 22.2×. The magnification can be switched between these two values by exchanging the tube lens (effl = 200 or 400 mm for 11.1×, 22.2×, respectively, when paired with an Olympus 10× objective, effl = 18 mm). In the former case, given a pixel size of 6.5 µm, the Nyquist-Shannon criterion provides a sampling-determined resolution limit of ca. 1.2 µm. Using the Rayleigh criterion for resolution *r*:

$$r = 0.61 \cdot \frac{\lambda_0}{NA}$$

And taking λ_0 in each case as the pass-band center (525, 845 nm respectively for GFP, AF800/CF800), the expected resolution assuming diffraction-limited performance at NA = 0.6 is ca. 534, 859 nm, respectively, both of which are below the Nyquist-Shannon criterion. Consequently, it is sampling rather than wavelength that determines the spatial resolution. However, in the latter case, the Nyquist-Shannon criterion provides a sampling-determined resolution limit of ca. 0.6 µm, which could allow diffraction-limited resolution for wavelengths > 590 nm and a substantial difference in resolution for GFP, AF800/CF800, respectively. The assumption made is that the imaging system performs in a diffraction-limited manner over the full wavelength range. The illumination system may be safely disregarded for consideration of lateral resolution; however, the imaging system formed by the imaging objective lens (Olympus XLPLNS10XSSVMP 10x/0.6, 8 mm WD) and ultra-broadband tube lens (Thorlabs, TTL200MP/AC508-400-AB-ML) may not provide diffraction-limited resolution, particularly the objective, which is optimized for two-photon imaging (most commonly ca. 920 nm). Given that axial chromatic aberrations are substantial for wavelengths < 700 nm, it is reasonable to also expect a decrease in the effective NA for shorter visible wavelengths and a convergence in the resolution for GFP and CF800/AF800. To explore whether this was apparent, bead stacks were analyzed using PSFj[1]. The lower bound for the lateral PSF FWHM for excitation/emission centers of 488/525 nm, 640/697 nm, 808/845 nm was found to be 741 ± 14, 797 ± 15, and 974 ± 18 nm, which are equivalent to maximum NAs of 0.44, 0.54, 0.54 respectively (from the Rayleigh criterion), demonstrating that the optical performance is best in the far-red - NIR as expected and approaches the theoretical value of 0.6. Although the lateral PSF for the GFP equivalent is slightly narrower, we note that this will be apparent only in extremely superficial regions. In any case, the difference is minor, and cell nuclei (ca. 5–10 µm), which are the smallest structural details that we sought to resolve, were easily resolvable for GFP, AF647, and CF800 in both sparsely labeled samples such as pescoids and Drosophila embryos, where the spacing between nuclei is substantially smaller (ca. 2–5 µm) as well as densely packed tissues, including the brain in the zebrafish larvae. The axial PSF FWHM is also similar for the three imaging bands: 5.46 ± 0.22, 5.36 ± 0.25 and 6.38 ± 0.41 µm for 488/525 nm, 640/697 nm, 808/845 nm. In the future, IR^2 could be combined with deconvolution strategies to improve the axial resolving power of multi-view light sheet microscopy as necessary[2].

Supplementary Note 2:

Fixation, permeabilization and staining strategies.

The deep learning-based restoration requires as close to 1:1 correspondence between i) the live and fixed state of the animal and ii) the distributions of GFP and the near-infrared dye therein post-staining. Several key challenges are apparent. Firstly, the fixation should maintain GFP fluorescence and not enhance autofluorescence. Secondly, the permeabilization step should allow antibody penetration but not distort or degrade the sample morphology. Thirdly, the staining step should allow permeation of the antibodies throughout the tissue to evenly stain without non-specific binding.

To ensure these requirements were met, various approaches for fixation, permeabilization, and tissue staining were tested and optimized for zebrafish samples. More generally, PFA fixation was found to be suitable for maintaining GFP fluorescence in all cases presented. However, the quenching of aldehydes via glycine washing reduced fixation-induced autofluorescence substantially. Various permeabilization steps were explored, including organic solvents (methanol/acetone), nonionic surfactants (Tween/Triton/DMSO), and proteinases (trypsin/proteinase K). Specifically, we tested reported protocols for zebrafish staining with slight modifications[3–5]. In some cases, antibody staining was lengthened to 7 days primary, 7 days secondary in an unsuccessful attempt to improve penetration. We found the standard protocol discussed in the methods section was sufficient for penetration of the vasculature label (Tg(kdrl:GFP)), best preserved the structure, and gave good labeling fidelity for a number of dyes. Nevertheless, the results from some dyes/affinity-tags were better than others. A large number of IR dye candidates were tested, and protocols utilizing primary antibodies only, primary and secondary antibodies, as well as nanobodies were assessed. Note that there are many important characteristics of a dye and affinity tag, such as brightness, photostability, excitation/emission maxima, solubility, specificity (IR dyes are typically large lipophilic molecules bearing several charged functional groups that offer water-solubility at a potential cost to specificity), staining time required, and completeness of staining. It was not possible to complete a full combinatorial assessment of all dyes and affinity tags independently, rather, we were limited by the commercially available options and cost constraints. The dyes, antibodies, and nanobodies tested that result in inferior (dimmer/less specific) staining than the case presented for the transgenic vasculature line Tg(kdrl:GFP) in Figure 1 are given in Supplementary Tables 1 and 2. We note, that other lines, permeabilization, and staining strategies or model organisms may provide better results with these products. None of the protocols attempted provided anything more than superficial penetration in the nuclear label (Tg(h2b:GFP) when used with antibody labeling. The custom-labeled CF800 nanobody (see Methods section), however, provided excellent staining in zebrafish and drosophila alike.

Supplementary Note 3:

Statistical comparison IR^2 vs N2V restored images.

To perform a more quantitative comparison of IR^2-restored with N2V-restored images, we set out to perform a patch-by-patch statistical analysis of the metrics obtained in Fig. 2. In particular, we analyzed patches for which the information content gain of IR images relative to the GFP were higher than 1, and bootstrapped the data: we randomly extracted 100 patches to avoid biasing the statistical analysis due to the large group size (N=2484 for zebrafish and N=9982 for Drosophila). Next, we overlaid line plots connecting metrics values for the same patch over IR, IR^2, and N2V images, and color-coded them in green for patches in which IR^2 values showed an improvement over IR and N2V and red for which IR^2 values were outperformed by N2V (Supplementary Figure 7).

For fish data, we observed that IR^2 patches had 38.0% more information content than their N2V counterpart. Overall, 71/100 patches had higher information content in the IR^2 than N2V. Under the null hypothesis that the N2V approach outperforms the IR^2 network, we performed a one-sided binomial test with number of trials = 100 and number of successes (IR^2>N2V) = 69, and obtained a statistic of 0.71, corresponding to a p-value = 1.6e-5. Therefore, we rejected the null hypothesis and instead showed that IR^2 outperforms the N2V approach. Similar values were obtained for SSIM and Pearson Correlation (Supplementary Table 3). Similarly, we also rejected the null hypothesis for Drosophila data with a p-value of 2.8e-7.

Supplementary Note 4:

IR-mSPIM spatial resolution in tissue.

The spatial resolutions reported for the IR-mSPIM microscope reported in Supplementary Note 1 are under the assumptions of negligible scattering and aberration arising from a weakly scattering fluorescent bead phantom and FEP tube/agarose gel, which are well-matched in refractive index to the surrounding water. The resolution in tissue is more difficult to define since it will be sample-dependent, and typically, tissue-derived images lack features that can unambiguously be assigned to single sub-diffraction sized emitters. As such, we use Image Decorrelation Analysis implemented in the NanoPyx python package [6,7], widely used in super-resolution microscopy to quantify spatial resolution on biological images. We applied the analysis on patches centered in the sample with regard to the two illumination directions and extracted at different depths into tissue (Supplementary Figure 2). When applied to deep tissue imaging, this measure cannot be interpreted as an absolute resolution of the microscope, but rather the smallest feature size that can be resolved under the conditions imposed by the light-tissue interaction. Considering the structures themselves do not vary in size with depth (nuclei/blood vessels), they thus provide a useful measure of the degradation of imaging performance in tissue and the depth to which various imaging strategies are able to resolve the underlying features. We observed that the feature size in IR-stained images is consistently lower than that of GFP images for even relatively superficial depths of a few tens of microns, supporting our quantification of image information content. Furthermore, this difference becomes more pronounced at increasing depth. This result suggests that

small features in images acquired with the IR-mSPIM can be better resolved than in the GFP images.

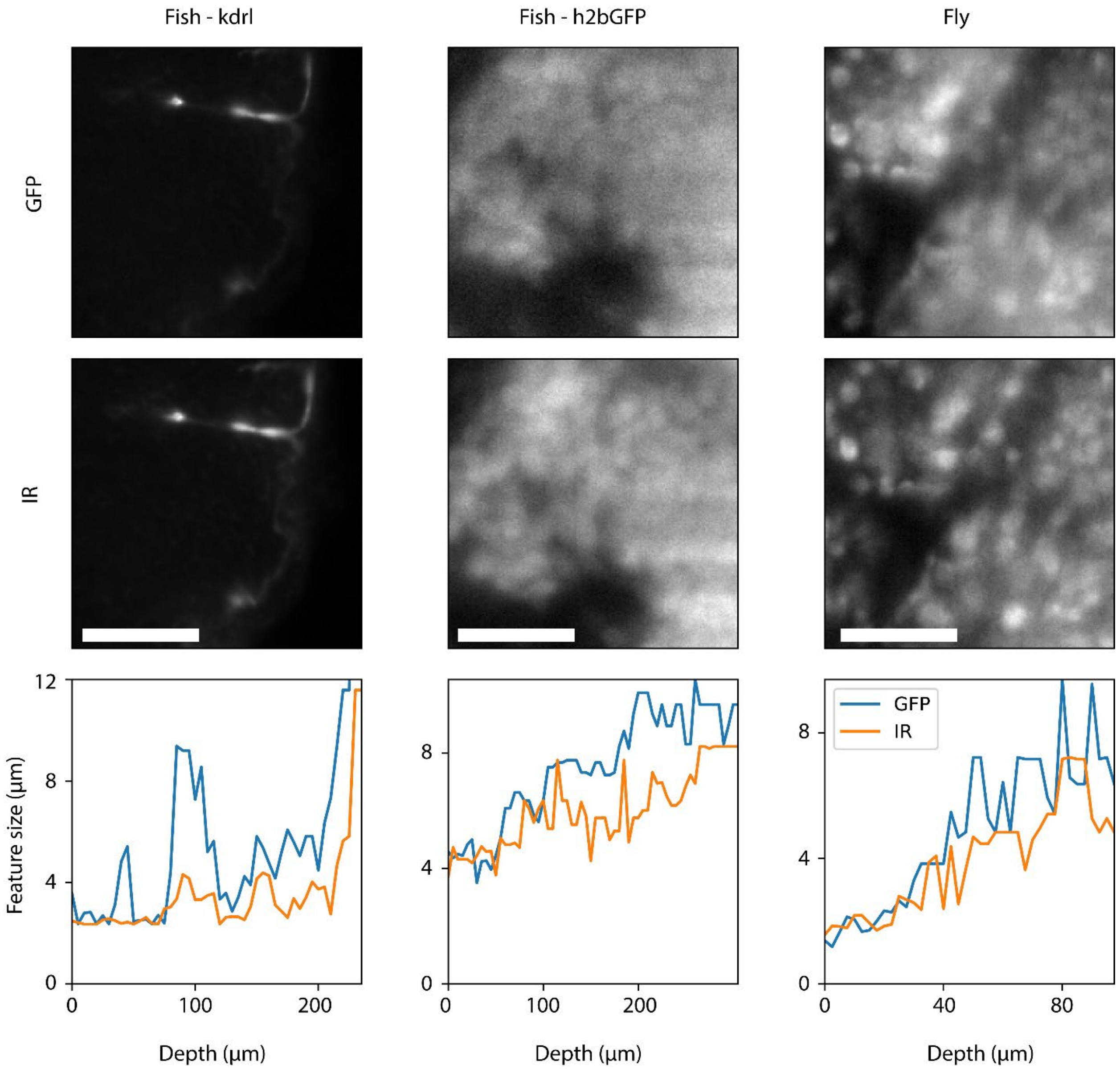

Supplementary Figure 2. Analysis of resolvable feature size. Top row: GFP, second row: IR-stained, third row: Resolvable feature size as a function of depth. Scale bars: 50 µm.

Supplementary Note 5:

Comparison of total effective laser exposure used for live imaging with light sheet fluorescence microscopy.

To explore whether the live imaging reported herein is performed within safe ranges of laser exposure for delicate developing embryos/larvae we must first consider the laser power at the sample. We used < 2.2 mW, <1.1 mW laser power at 488 nm for live imaging

of zebrafish and drosophila larva/embryos, respectively. For approximately equivalent light-sheet imaging schemes, the photon burden experienced by the live sample can be described by the laser power summed for the total number of exposures over the duration of the experiment. For the zebrafish (ca. 72 hrs, dt = 300 s, one color channel) and drosophila (ca. 24 hrs, dt = 300 s, one color channel) data presented, we calculate 1,848 and 275 mW equivalent exposures, respectively. For comparison, Schmid et al. used 17,280 mW equivalent exposures for early imaging of zebrafish embryogenesis (6 mW, ca. 12 hrs, dt = 30 s, one color channel) [8]. Shah et al. used 13,824 mW for conceptually similar imaging (8 mW, ca. 12 hrs, dt = 150 s, three color channels) [9]. Weber et al. used 2 mW at the illumination objective back aperture (ca. 1.6 mW at the object assuming 80% transmission through the objective lens), which was lower than the 5 mW (4 mW at the object) threshold for a measurable increase in heart rate in the zebrafish embryo/larva [10]. Chhetri et al. performed developmental imaging of drosophila using 2,160 mW equivalent exposures (0.1 mW, ca. 3 hrs, dt = 4 s, two color channels). In this case since the light sheet was produced temporally by line scanning, the instantaneous or peak intensity is far higher than the other examples, or indeed the studies presented herein, making direct comparison difficult [11]. Despite the challenges in directly comparing the different studies, we note that the <2.1 mW laser power used at 488 nm for zebrafish is below the threshold shown by Weber et al. which is below the threshold shown by Weber et al. for perturbation of the heart beat period. The mW equivalent exposures being substantially lower than the and substantially below the laser power usage of Schmid and Shah et al. For live imaging of zebrafish/drosophila, we used a maximum laser power at source of 17.2/8.6 mW resulting in a maximum power at the sample of 2.2/1.1 mW, for single-color live imaging from a single illumination view (presented data). For the zebrafish (ca. 72 hrs, every 300 s) and drosophila (ca. 24 hrs every 300 s) data presented, = 1,848 and 275 mW equivalent exposures respectively, lower than all other examples considered other examples considered demonstrates that the photon burden associated with live imaging presented is well within a normal range for live imaging of developing embryos and well within ranges of exposure considered safe for long term live imaging.

Supplementary Table 1. Combination of primary, secondary antibodies and dyes used throughout.

Primary	Manufacturer (Cat. number)	Secondary	Manufacturer (Cat. number)	Notes	Fig.
aGFP	ThermoFisher (A-11122)	AF800	ThermoFisher (A-32808)	Primary-secondary incubation	Fig. 1 (kdrl:GFP), Sup. Fig. 2
nGFP		AF647	Chromotek (GB2AF647)	Commercially conjugated	Sup. Fig. 2
nGFP	Chromotek (GT-250)	CF800	Biotium (#92128)	Custom conjugated	Fig. 1 (h2b:GFP), Fig. 2, Fig. 3, Fig. 4
nGFP	Chromotek (GT-250)	AF700	ThermoFisher (A-21038)	Custom conjugated	Sup. Fig. 1

Supplementary Table 2. Image metrics relative to Figure 2.

		GFP		IR		IR^2		N2V	
		Mean	SD	Mean	SD	Mean	SD	Mean	SD
Fish data	Pearson corr.	0.900	0.144	NA	NA	0.921	0.117	0.824	0.152
	Info content gain	NA	NA	1.228	0.213	1.096	0.198	0.973	0.338
	SSIM	0.865	0.071	NA	NA	0.929	0.046	0.766	0.097
Fly data	Pearson corr.	0.820	0.153	NA	NA	0.750	0.212	0.531	0.442
	Info content gain	NA	NA	1.627	0.951	1.466	1.189	1.466	1.189
	SSIM	0.881	0.053	NA	NA	0.911	0.050	0.771	0.160

Supplementary Table 3. Patch-by-Patch statistical analysis.

		N patches analyzed	% improv. IR^2 over N2V	IR^2 better than N2V	P-value (1-sided binomial test)
Fish data	Info content gain	100	22.2	71	1.6e-5
	SSIM	100	22.7	100	7.9e-31
	Pearson correlation	100	13.8	99	8.0e-29
Fly data	Info content gain	100	74.4	75	2.8e-7
	SSIM	100	35.5	98	4.0e-27
	Pearson correlation	100	9.2	82	3.1e-11

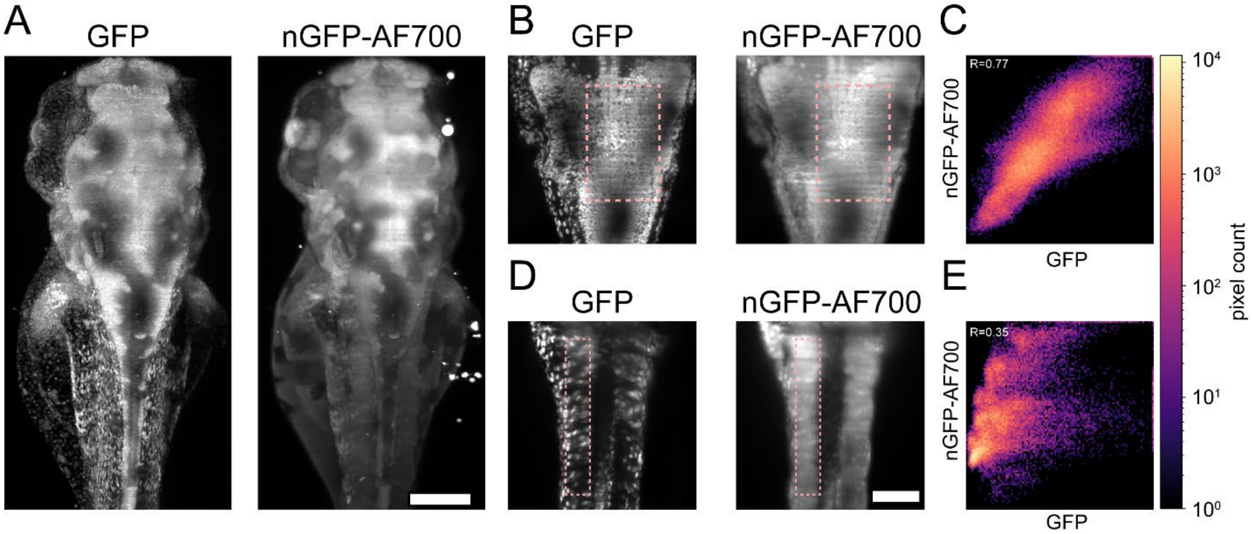

Supplementary Figure 3. A) Maximum intensity projection of a Tg(kdrl:GFP) zebrafish larva at 3 days post fertilization, stained using a nanobody conjugated to a dye (Alexa Fluor 700) in the NIR regime. B, D) Maximum intensity projection of a sub-stack of the same fish in panel A) in the brain (B) and tail (D) region. C, E) Pixel-wise Pearson correlation coefficient for the regions highlighted in panels B and D. Scale bars: 100 µm.

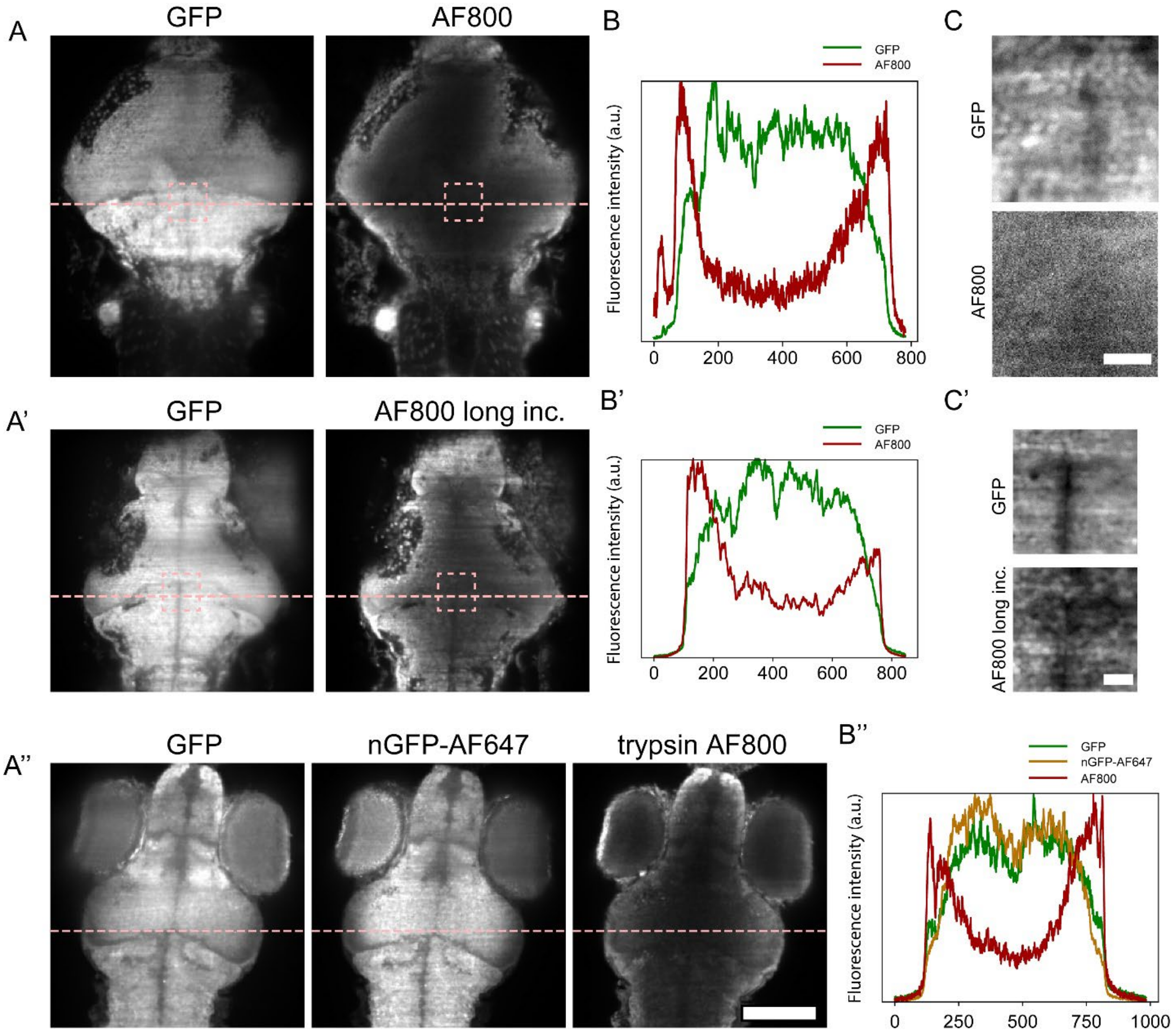

Supplementary Figure 4. A,A',A'') Single focal plane images from Tg(h2b::GFP) zebrafish stained with conventional primary-secondary antibody protocol (A), antibody protocol using 7 days incubation period (A') and simultaneous nanobodies and trypsin-based antibodies staining protocols (A''). Scale bar: 100 µm. B, B', B'') Line profile across the center of the brain region for the images shown in Panel (A) (Dashed pink lines). C, C') Zoomed in visualization of the deepest region in the brain from the images shown in Panel (A) (Dashed pink boxes). Scale bar: 5 µm.

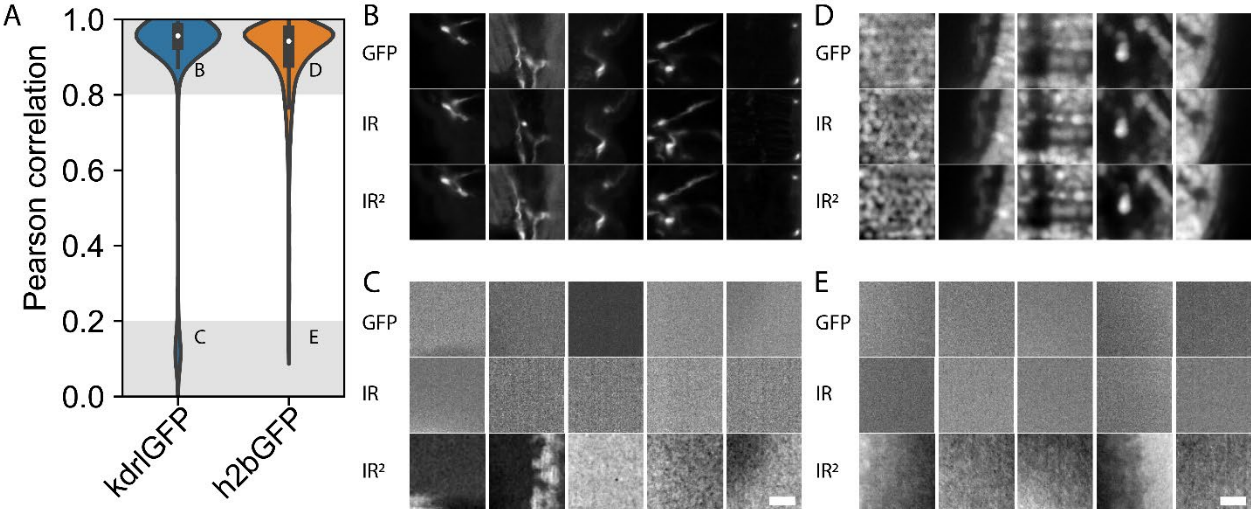

Supplementary Figure 5. A) Pearson correlation coefficient as shown in Figure 1 panel D. Inner box plots are drawn around the mean and first and third quartiles. White dots represent mean values. B D) Patches examples with high correlation coefficients extracted from images of kdrl:GFP and h2b:GFP fish larvae, respectively. Scale bar: 5 μm. C, E) Patches examples with low correlation coefficients, extracted from images of kdrl:GFP and h2b:GFP fish larvae, respectively. Scale bar: 5 μm.

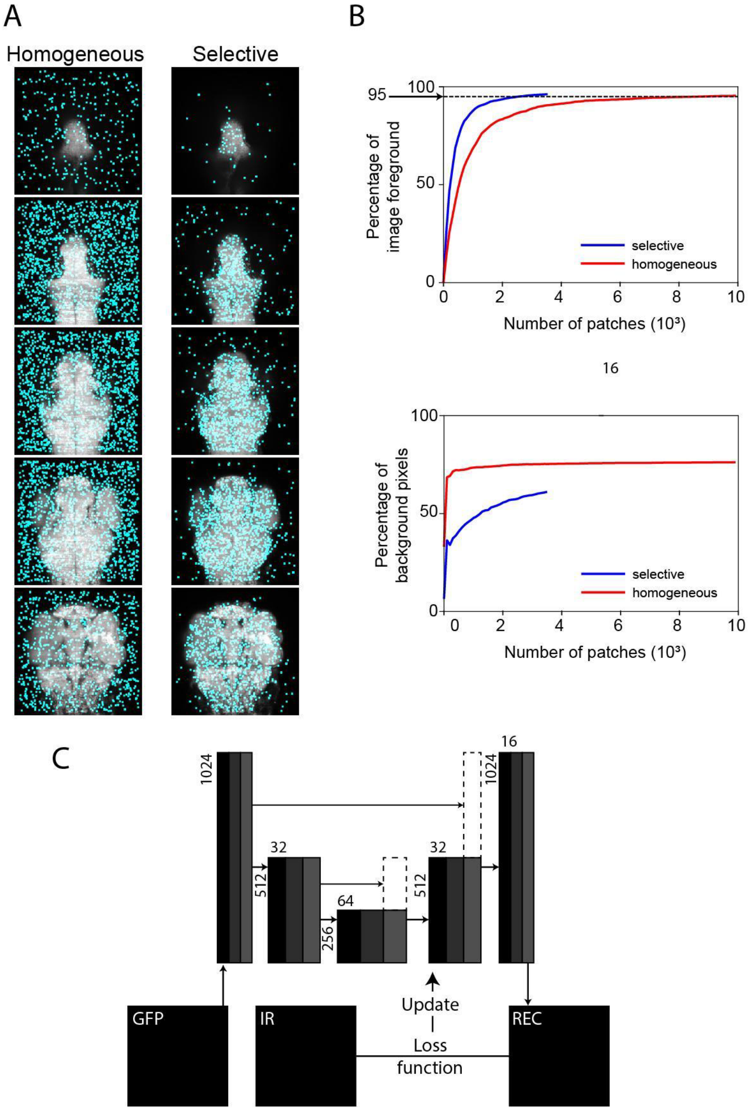

Supplementary Figure 6. A) Single *z*-planes of a fixed Tg(h2b:GFP) zebrafish larva (72 hpf). The transparent light blue squares represent the patches extracted from the sample for network training. B) Fraction of sample coverage (top) and percentage of background pixels in the training set (bottom) with an increasing number of patches. Red and blue lines represent the fraction of pixels in high and low information content regions, respectively. C) A schematic of the U-Net deep learning network used for restoration[12]. Horizontally and vertically aligned numbers denote the channel and spatial dimensions of the images at every layer of the U-Net architecture.

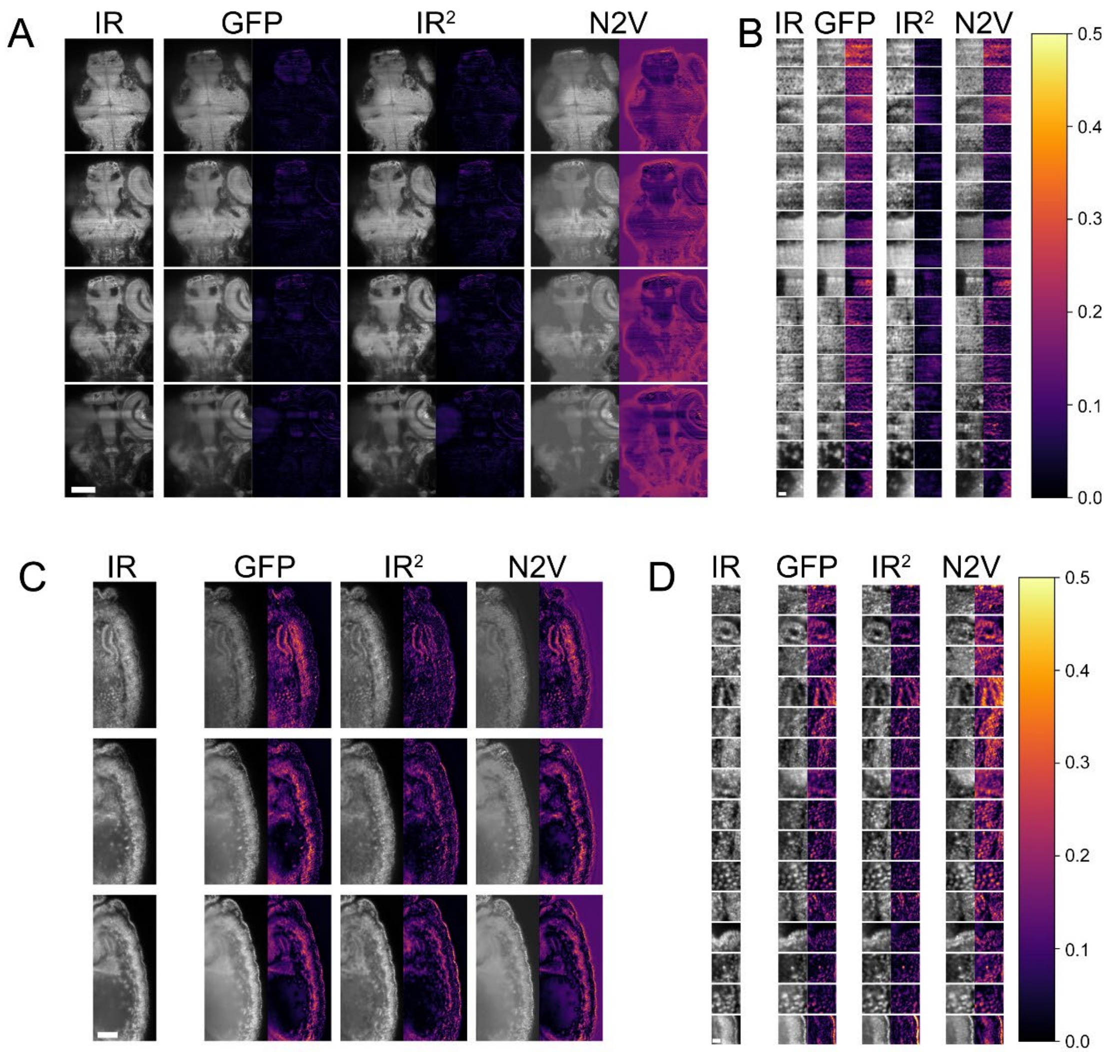

Supplementary Figure 7. A) IR, GFP, IR^2- and N2V-restored images extracted at increasing detection depth in a 96 hpf Tg(h2b:GFP) zebrafish larva, shown side by side with their respective difference map relative to the ground truth IR image. Scale bar: 100 µm **B)** Example patches and relative difference maps for the same zebrafish sample shown in A) arranged for increasing average normalized root mean squared error in the IR^2 patch. Scale bar: 5 µm. **C)** IR, GFP, IR^2- and N2V-restored images extracted at increasing detection depth in a 8 hpf Tg(His2AV-GFP) drosophila larva, shown side by side with their respective difference map relative to the ground truth IR image. Scale bar: 100 µm **D)** Example patches and relative difference maps for the same drosophila sample shown in A) arranged for increasing average normalized root mean squared error in the IR^2 patch. Scale bar: 5 µm.

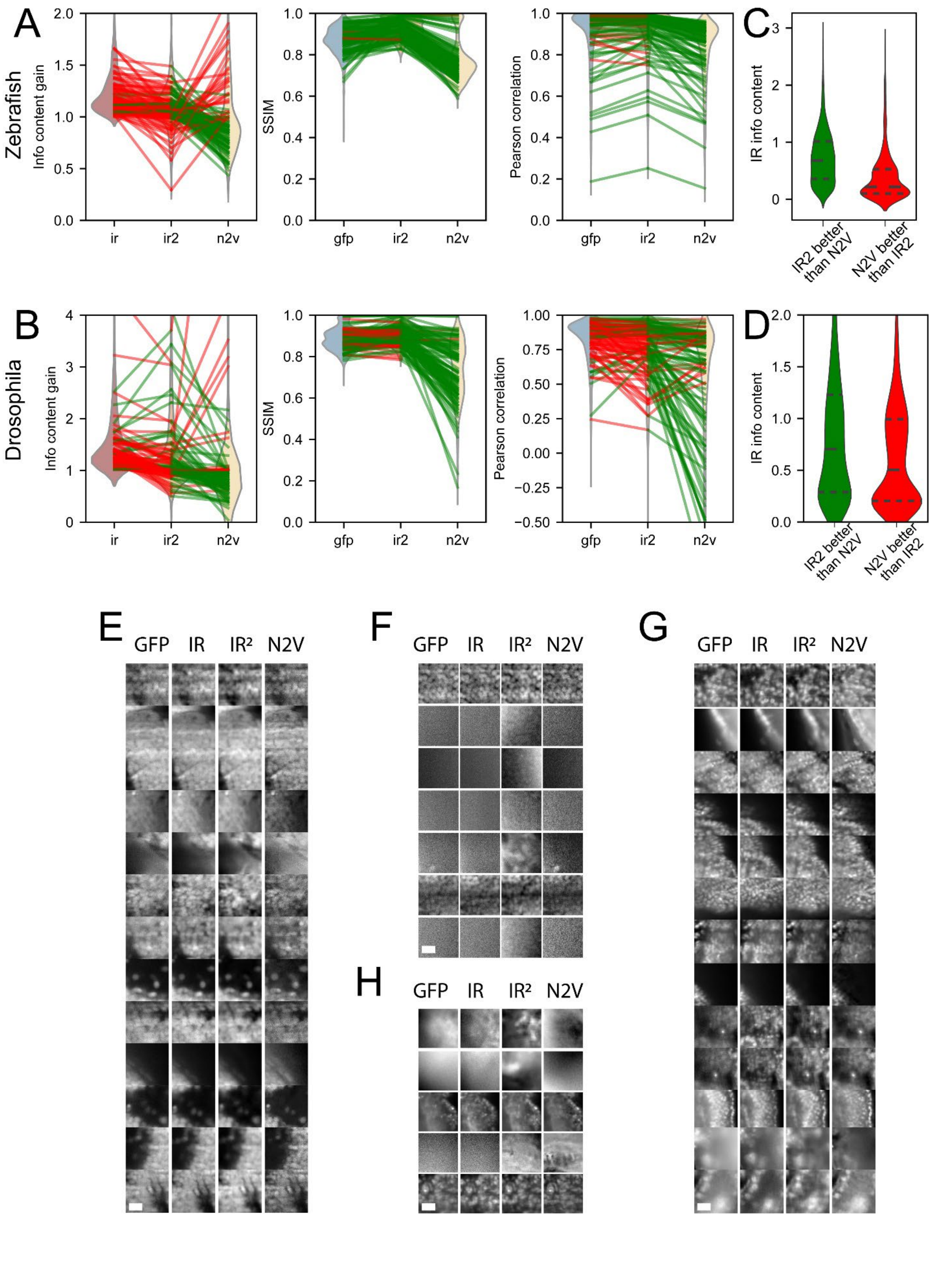

A
B
C
D
E
F
G
H
Zebrafish
Drosophila
Info content gain
SSIM
Pearson correlation
IR info content
ir
ir2
n2v
gfp
IR2 better than N2V
N2V better than IR2
GFP
IR
IR²
N2V

Supplementary Figure 8. Quantitative comparison of individual patches between IR, IR^2 and N2V for zebrafish (A) and Drosophila samples (B). Underlying violin plots are the same as in Fig2. Overlaid lines connect metrics across approaches for the same patch, and are color-coded according to the performance of IR^2 with the connecting approach (red= IR^2 worse, green= IR^2 better). C), D) Distribution of absolute information content in the ground truth IR images for patches in which IR^2 outperforms N2V (green) or vice versa (red). Dash lines represent 25/50/75th quartiles. E), G) Example patches for which IR^2 performs better than N2V in terms of information content gain in zebrafish and drosophila samples, respectively. Scale bar: 5 μm. F), H) Example patches for which N2V performs better than IR^2 in terms of information content gain in zebrafish and drosophila samples, respectively. Scale bar: 5 μm.

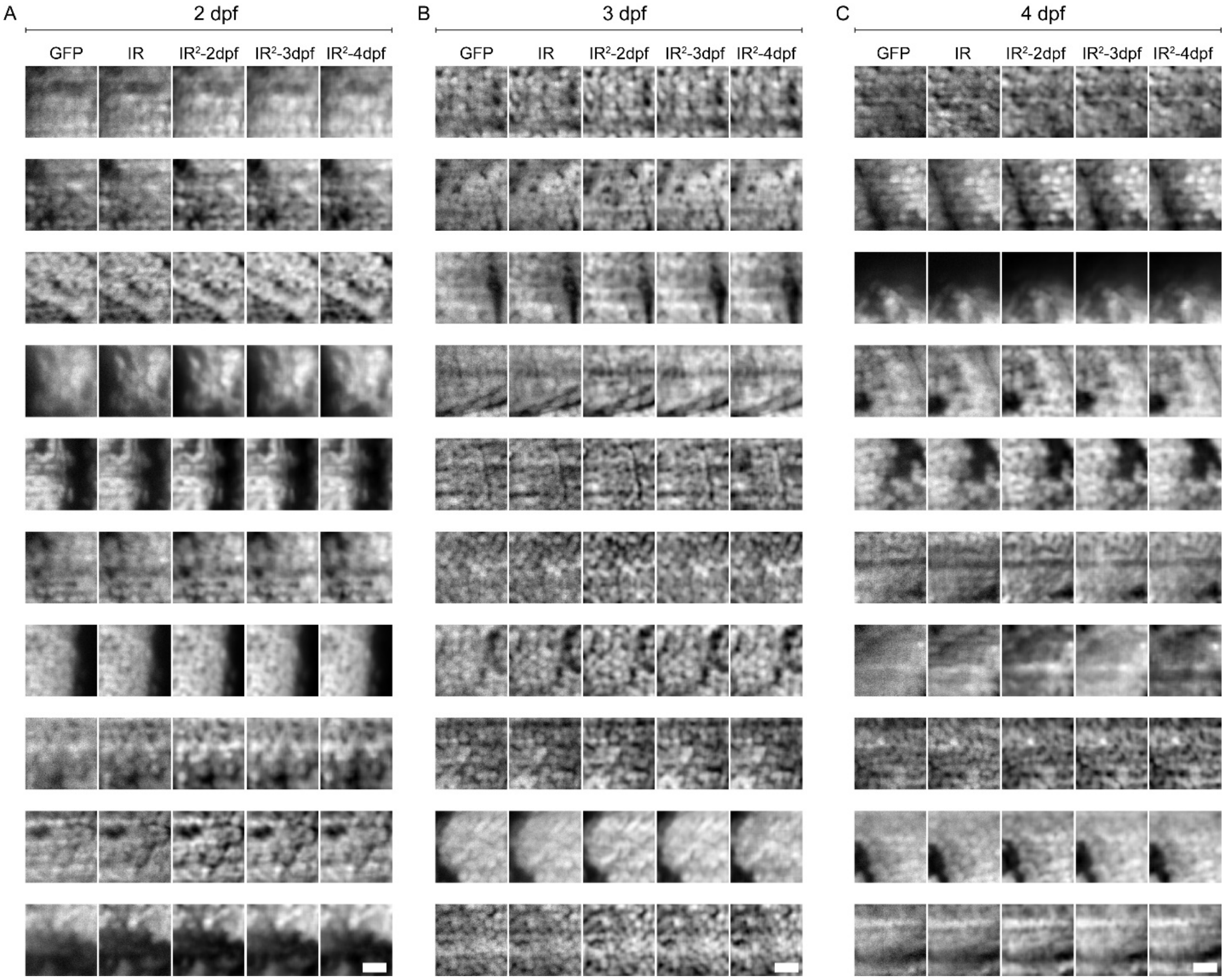

Supplementary Figure 9. Example patches from fish larvae images at 2 (A), 3 (B) and 4 (C) days post fertilization. First columns: input image (endogenous GFP), second column: near infrared image, remaining columns: patches restored using models trained from fish at 2 (third column), 3 (fourth column) and 4 (fifth column) days post fertilization. Scale bar: 5 µm.

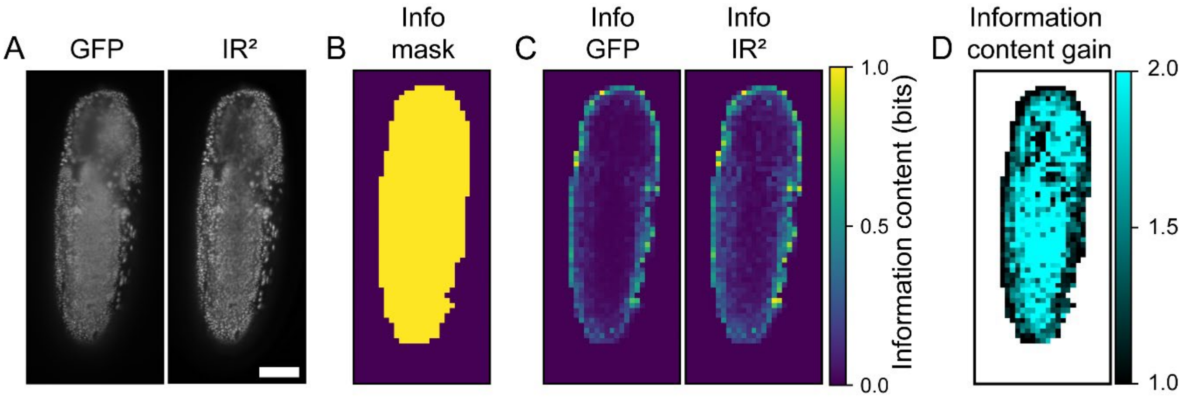

Supplementary Figure 10. Information content gain pipeline. A) Endogenous GFP and near-infrared individual Z-planes of a drosophila larvae used as input images. Scale bar: 100 µm. B) Binary mask obtained after automated thresholding. C) Absolute information content in the binary mask image, obtained using a sliding window. CD) Information content gain for the images shown in A), defined as the ratio between the information content of the images shown in C) (see Methods for details).

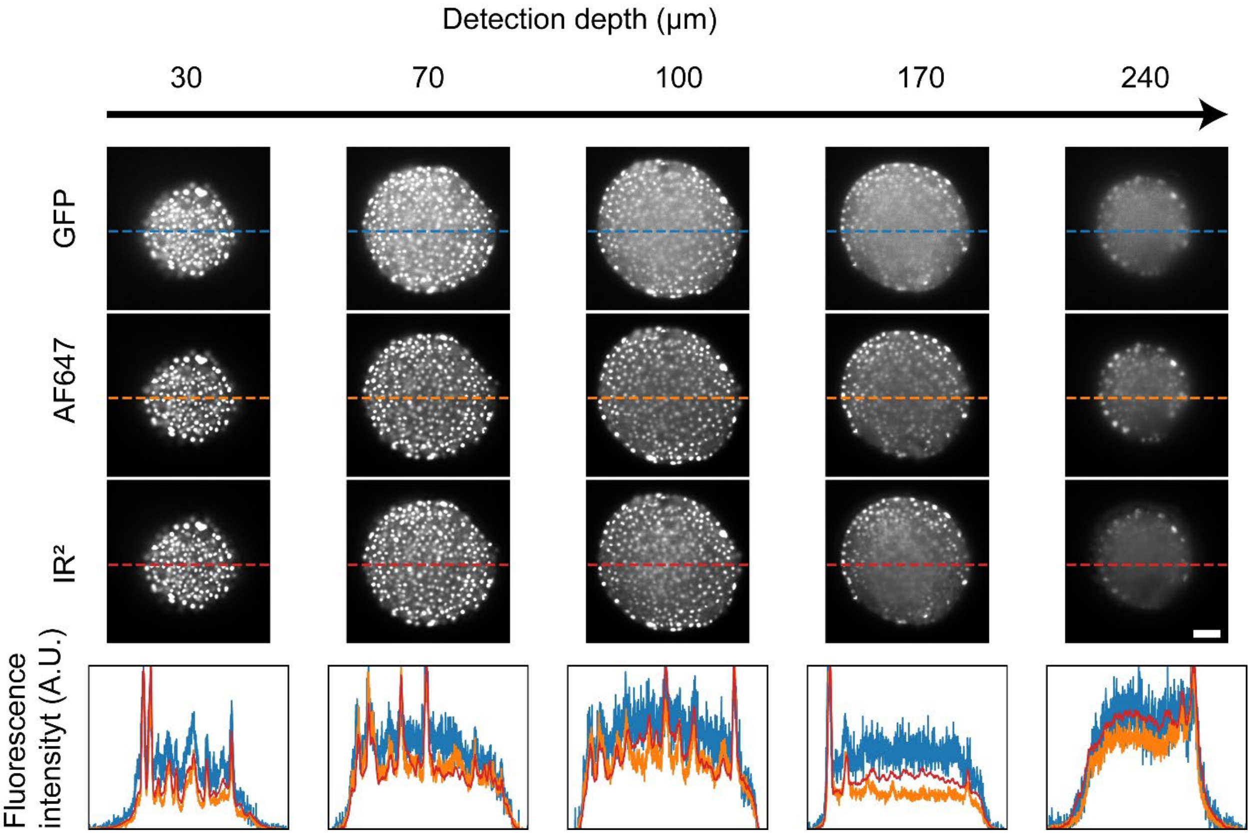

Supplementary Figure 11. Images of a pescoids stained with a polyclonal antibody against GFP, and conjugated with Alexa Fluor 647 at increasing detection depths and restored using a dedicated pescoid IR^2 model. Top row: GFP, second row: AF647, third row: IR^2 images. Images are shown after normalization using their 3rd and 99.7th percentiles. Scale bar: 50 µm. Bottom panels: plot profiles along the colored dashed lines depicted in the images (blue: GFP, orange: AF647, red: IR^2).

Supplementary Movie 1. 3D reconstruction of Drosophila embryo development. Top panels: GFP, bottom panels: IR^2. Left panels: XY view, central panels: XZ view (rotated by 90 degrees around the Z axis), right panels: single Z plane approximately at the center of the embryo. Scale bar: 100 µm.

Supplementary Movie 2. 3D reconstruction of early pescoid (first image of the time lapse). Top panel: GFP, bottom panel: IR^2. Scale bar: 50 µm.

Supplementary Movie 3. 3D reconstruction of pescoid development over the whole 10 hours of the time lapse experiment. Top panel: GFP, bottom panel: IR^2. Scale bar: 50 µm.

References

1. Theer, P., Mongis, C. & Knop, M. PSFj: know your fluorescence microscope. *Nat. Methods* **11**, 981–982 (2014).
2. Schmid, B. & Huisken, J. Real-time multi-view deconvolution. *Bioinformatics* **31**, 3398–3400 (2015).
3. Hunter, P. R. *et al.* Localization of Cadm2a and Cadm3 proteins during development of the zebrafish nervous system. *J. Comp. Neurol.* **519**, 2252–2270 (2011).
4. Inoue, D. & Wittbrodt, J. One for All—A Highly Efficient and Versatile Method for Fluorescent Immunostaining in Fish Embryos. *PLoS ONE* vol. 6 e19713 Preprint at https://doi.org/10.1371/journal.pone.0019713 (2011).
5. Lindsey, B. W. *et al.* The role of neuro-epithelial-like and radial-glial stem and progenitor cells in development, plasticity, and repair. *Prog. Neurobiol.* **170**, 99–114 (2018).
6. Descloux, A., Grußmayer, K. S. & Radenovic, A. Parameter-free image resolution estimation based on decorrelation analysis. *Nat. Methods* **16**, 918–924 (2019).
7. Saraiva, B. M. *et al.* NanoPyx: super-fast bioimage analysis powered by adaptive machine learning. *bioRxiv* 2023.08.13.553080 (2023) doi:10.1101/2023.08.13.553080.
8. Schmid, B. *et al.* High-speed panoramic light-sheet microscopy reveals global endodermal cell dynamics. *Nat. Commun.* **4**, 2207 (2013).
9. Shah, G. *et al.* Multi-scale imaging and analysis identify pan-embryo cell dynamics of germlayer formation in zebrafish. *Nat. Commun.* **10**, 5753 (2019).
10. Weber, M. *et al.* Cell-accurate optical mapping across the entire developing heart. *Elife* **6**, (2017).
11. Chhetri, R. K. *et al.* Whole-animal functional and developmental imaging with isotropic spatial resolution. *Nat. Methods* **12**, 1171–1178 (2015).
12. Ronneberger, O., Fischer, P. & Brox, T. U-Net: Convolutional Networks for Biomedical Image Segmentation. *Lecture Notes in Computer Science* 234–241 Preprint at https://doi.org/10.1007/978-3-319-24574-4_28 (2015).
