# Supplementary material for: Image restoration of degraded time-lapse microscopy data mediated by near-infrared imaging
Source: Nat Methods. 2024 Jan 4;21(2):311–21. doi: 10.1038/s41592-023-02127-z (PMC10864180; doi:10.1038/s41592-023-02127-z)
Supplement: Supplementary file 2 — Reporting Summary [file 41592_2023_2127_MOESM2_ESM.pdf]

nature portfolio

Corresponding author(s): Huisken, Jan

Last updated by author(s): Nov 16, 2022

Reporting Summary

Nature Portfolio wishes to improve the reproducibility of the work that we publish. This form provides structure for consistency and transparency in reporting. For further information on Nature Portfolio policies, see our Editorial Policies and the Editorial Policy Checklist.

Statistics

For all statistical analyses, confirm that the following items are present in the figure legend, table legend, main text, or Methods section.

n/a	Confirmed	
☐	☒	The exact sample size (*n*) for each experimental group/condition, given as a discrete number and unit of measurement
☐	☒	A statement on whether measurements were taken from distinct samples or whether the same sample was measured repeatedly
☐	☒	The statistical test(s) used AND whether they are one- or two-sided<br>*Only common tests should be described solely by name; describe more complex techniques in the Methods section.*
☒	☐	A description of all covariates tested
☒	☐	A description of any assumptions or corrections, such as tests of normality and adjustment for multiple comparisons
☐	☒	A full description of the statistical parameters including central tendency (e.g. means) or other basic estimates (e.g. regression coefficient) AND variation (e.g. standard deviation) or associated estimates of uncertainty (e.g. confidence intervals)
☐	☒	For null hypothesis testing, the test statistic (e.g. *F*, *t*, *r*) with confidence intervals, effect sizes, degrees of freedom and *P* value noted<br>*Give P values as exact values whenever suitable.*
☒	☐	For Bayesian analysis, information on the choice of priors and Markov chain Monte Carlo settings
☒	☐	For hierarchical and complex designs, identification of the appropriate level for tests and full reporting of outcomes
☐	☒	Estimates of effect sizes (e.g. Cohen's *d*, Pearson's *r*), indicating how they were calculated

Our web collection on statistics for biologists contains articles on many of the points above.

Software and code

Policy information about availability of computer code

Data collection	For data acquisition, the microscope was controlled in a LabVIEW environment (NI LabVIEW 2017) using custom software, NI multi-function DAQ hardware (NI DAQmx) and various hardware SDKs. Images acquired with Luxendo/Bruker MuVi SPIM microscope were pre-processed with Luxendo software v3.0.
Data analysis	The Python code used isavailable at https://github.com/grinic/2023_InfraRed_Image_Restoration.git. A sample dataset is available on the Zenodo repository (doi 10.5281/ zenodo.7075414). For training and prediction of deep learning networks, we used the CARE Python3 package with Tensorflow v.2.5.0. Image pro

For manuscripts utilizing custom algorithms or software that are central to the research but not yet described in published literature, software must be made available to editors and reviewers. We strongly encourage code deposition in a community repository (e.g. GitHub). See the Nature Portfolio guidelines for submitting code & software for further information.

Data

Policy information about availability of data

All manuscripts must include a data availability statement. This statement should provide the following information, where applicable:

- Accession codes, unique identifiers, or web links for publicly available datasets
- A description of any restrictions on data availability
- For clinical datasets or third party data, please ensure that the statement adheres to our policy

A sample of the dataset used is available on Zenodo (doi 10.5281/zenodo.7075414). Due to the large size of the full dataset (in the order of several TB) and the 50GB limit of Zenodo, we will make the full dataset available upon request.

Human research participants

Policy information about studies involving human research participants and Sex and Gender in Research.

Reporting on sex and gender	No human participants were involved in the study
Population characteristics	No human participants were involved in the study
Recruitment	No human participants were involved in the study
Ethics oversight	No human participants were involved in the study

Note that full information on the approval of the study protocol must also be provided in the manuscript.

Field-specific reporting

Please select the one below that is the best fit for your research. If you are not sure, read the appropriate sections before making your selection.

☒ Life sciences ☐ Behavioural & social sciences ☐ Ecological, evolutionary & environmental sciences

For a reference copy of the document with all sections, see nature.com/documents/nr-reporting-summary-flat.pdf

Life sciences study design

All studies must disclose on these points even when the disclosure is negative.

Sample size	For each of the model system used, we analyzed 3-5 fixed/stained samples. We did not use a statistical test to calculate sample size,, instead the sample size was empirically determined based on the replicates needed for the deep learning approach. Typically, one sample was used for network training and the other samples for testing and prediction. For the time-lapse microscopy dataset, we used 1 sample per model system as a proof-of-principle.
Data exclusions	No data were excluded.
Replication	Samples were randomly chosen from a pool of samples laid on the day of the experiments. Data were acquired on multiple days and for different sample batches over the course of several months. All attempts of replication were successful.
Randomization	From the pool of zebrafish and drosophila samples, individuals for imaging were chosen randomly. Samples were allocated into experimental group according to their developmental stage. The first acquired sample in each category was generally used to train the deep learning network, and the others were used for testing and prediction.
Blinding	Blinding was not relevant to this study due to the randomization used and no categorization of the data.

Reporting for specific materials, systems and methods

We require information from authors about some types of materials, experimental systems and methods used in many studies. Here, indicate whether each material, system or method listed is relevant to your study. If you are not sure if a list item applies to your research, read the appropriate section before selecting a response.

Materials & experimental systems

n/a	Involved in the study	
☐	☒	Antibodies
☒	☐	Eukaryotic cell lines
☒	☐	Palaeontology and archaeology
☐	☒	Animals and other organisms
☒	☐	Clinical data
☒	☐	Dual use research of concern

Methods

n/a	Involved in the study	
☒	☐	ChIP-seq
☒	☐	Flow cytometry
☒	☐	MRI-based neuroimaging

Antibodies

Antibodies used	Primary: anti-GFP (ThermoFisher, A-11122), nano-GFP (Chromotek, GT-250)<br>Secondary: AF800 (ThermoFisher, A-32808), CF800 (Biotium, #92128), AF700 (TermoFisher, A-21038)<br>Conjugated: nano-GFP+AF647 (Chromotek, GB2AF647)
Validation	Each antibody was validated by the commercial company providing the product.

Validation

The primary anti-GFP has been used extensively for immunohistochemistry in various model systems such as drosophila, zebrafish and mouse. Relevant literature can be found at: https://www.thermofisher.com/antibody/product/GFP-Antibody-Polyclonal/A-11122.

For the nano-GFP (GT-250), relevant literature can be found at:
https://www.ptglab.com/products/GFP-VHH-recombinant-binding-protein-gt.htm#publications,
and Chromotek provides the following statements:
Alpaca anti-GFP VHH, purified recombinant binding protein for extraordinary stable & reliable binding
• GFP-VHH:GFP complex is stable up to 80 °C, 1 mM DTT, 3 M Guanidinium•HCl, 8 M Urea, 2 M NaCl, 2 % Nonidet P40 Substitute, 1 % SDS, 1 % Triton X-100, 3 % Deoxycholate
• Fulfills highest requirements for antibody validation
• Structure and function are characterized

For the conjugated nanobody (GB2AF647), relevant literature can be found at:
https://www.ptglab.com/products/GFP-Booster-Alexa-Fluor-647-gb2AF647.htm
Chromotek provides the following description:
The GFP-Booster stabilizes, enhances, and reactivates the signal of GFP-fusion proteins. Due to its small size, the GFP-Booster enables higher image quality in epifluorescence, confocal, and super-resolution microscopy:
• Considerably higher tissue penetration rates
• Superior accessibility and labelling of epitopes in crowded cellular/organelle environments
• Less than 2 nm epitope-label displacement minimizes linkage error
• Monovalent VHHs do not cluster their epitopes
• Validation: structure and function characterized
• Consistent and reliable performance due to recombinant production

Animals and other research organisms

Policy information about studies involving animals; ARRIVE guidelines recommended for reporting animal research, and Sex and Gender in Research

Laboratory animals	Zebrafish: transgenic lines Tg(kdrl:GFP) and Tg(h2b:GFP) at larval stages between 24 and 144 hours post fertilization.<br>Drosophila: Tg(his2av:GFP) at embryo stages between 4 and 24 hours post fertilization.
Wild animals	The study did not invlove wild animals.
Reporting on sex	The study does not apply to one sex only.
Field-collected samples	The study did not include samples collected from the field.
Ethics oversight	Zebrafish (Danio rerio) were handled according to established protocols approved by the 380 University of Wisconsin-Madison Animal Care and Use Committee.<br>Fly stocks were maintained by the lab of Jill Wildonger at the University of Wisconsin-Madison 420 according to established protocols approved by the University of Wisconsin- Madison Animal 421 Care and Use Committee.

Note that full information on the approval of the study protocol must also be provided in the manuscript.
